# Five-Year Slaughterhouse-Based Surveillance of *Echinococcus granulosus* in Sheep from Yili, Northwest Xinjiang, China

**DOI:** 10.3390/pathogens15010040

**Published:** 2025-12-29

**Authors:** Cairen, Xiaoli Zhang, Li Zhang, Kalibixiati Aimulajiang, Batubayier Daoerji, Daoerji Namuka, Baoping Guo, Rongsheng Mi, Liying Wang

**Affiliations:** 1 NHC Key Laboratory of Parasite and Vector Biology, National Institute of Parasitic Diseases, Chinese Center for Disease Control and Prevention (Chinese Center for Tropical Diseases Research), Shanghai 200025, China; m18699138331@163.com; 2 State Key Laboratory of Pathogenesis, Prevention and Treatment of High Incidence Diseases in Central Asia, Xinjiang Medical University, Urumqi 830054, China; kali0920@163.com (K.A.); m13209967331@163.com (B.D.); cai-mike@163.com (D.N.); 3 WHO-Collaborating Centre for Prevention and Care Management of Echinococcosis, The First Affiliated Hospital of Xinjiang Medical University, Urumqi 830054, China; 4 Jiangsu Agri-Animal Husbandry Vocational College, Taizhou 225300, China; 2024010600@jsahvc.edu.cn; 5 Key Laboratory of Forensic Medicine, School of Basic Medical Sciences, Xinjiang Medical University, Urumqi 830011, China; tianyemengxing@126.com; 6 Key Laboratory of Animal Parasitology of Ministry of Agriculture, Laboratory of Quality and Safety Risk Assessment for Animal Products on Biohazards (Shanghai) of the Ministry of Agriculture, Shanghai Veterinary Research Institute, Chinese Academy of Agricultural Sciences, Shanghai 200241, China

**Keywords:** cystic echinococcosis, prevalence, temporal variation, morphological, genotype, haplotype

## Abstract

Background: Cystic echinococcosis (CE) remains a significant zoonotic burden in the pastoral regions of China. Yili Prefecture in Xinjiang is a high-risk area, yet comprehensive data are lacking on the prevalence and molecular characteristics of *Echinococcus granulosus* in its primary intermediate host, sheep. Methods: From 2020 to 2024, a total of 2700 sheep livers were visually inspected for hydatid cysts infection at one randomly selected slaughterhouse in each of the nine counties of Yili Prefecture. Ninety cyst-positive samples were subjected to morphological examination and molecular genotyping by amplifying and sequencing the *nad2* gene. Results: The overall prevalence of *E. granulosus* was 22.0% (594/2700). County-level prevalence ranged from 18.3% (Zhaosu County) to 25.7% (Huocheng County), with no significant differences observed among the counties (*p* > 0.05) except between Huocheng and Zhaosu. Temporally, the annual prevalence fluctuated between 20.2% and 24.2% without a consistent downward trend. Genotyping revealed that the G1 genotype was overwhelmingly dominant (95.2%, 79/83), with a minor circulation of the G3 genotype (4.8%, 4/83). Fourteen haplotypes were identified; Hap1 was the central and predominant haplotype (47.0%, 39/83), found in all counties. Network analysis suggested a recent population expansion of the parasite. Conclusion: This five-year surveillance study reveals a persistently high prevalence and complex genetic diversity of *E. granulosus* in sheep in Yili Prefecture. The dominance of the zoonotic G1 genotype indicates a substantial public health threat. Our findings provide crucial data for contributing to the development of local control strategies. However, the specific reasons for the high infection rate in sheep remain unclear, as this study did not include examinations of definitive hosts or environmental samples; this gap should be addressed in future research.

## 1. Introduction

*Echinococcus granulosus* is a zoonotic cestode whose larval stage is the causative agent of cystic echinococcosis (CE), a severe parasitic disease affecting both livestock and humans [1]. The parasite has a global distribution, with endemic foci predominantly located in the Northern Hemisphere, including extensive regions of Asia, Europe, and North America [2,3]. In these endemic areas, the annual human incidence of CE ranges from less than 1 to 200 cases per 100,000 individuals [4]. This disease imposes a substantial burden on public health, accounting for an estimated global loss of 285,500 disability-adjusted life years annually (DALYs) [5,6]. China has an estimated 30 million livestock infected with CE and 7 million new annual infections, over 70% of which occur in sheep. These infections result in annual economic losses of approximately 1 billion CNY (RMB), primarily due to decreased body weight, reduced milk production, and deteriorated meat and wool quality [7]. In response to its significant impact, the World Health Organization (WHO) has classified echinococcosis as a neglected tropical disease (NTD) [8]. Since 2005, China has intensified its national echinococcosis control efforts through central government funding and policies, with the goal of controlling and eliminating the disease by 2030 [8].

China is a highly endemic area for both alveolar echinococcosis (AE) and CE, with CE having a higher prevalence [9]. The disease is hyperendemic in western and northwestern pastoral regions [10]. Humans and livestock acquire *E. granulosus* infection by ingesting eggs from environments contaminated by feces of infected definitive hosts (e.g., dogs) [11]. Following ingestion, the released oncospheres form metacestode cysts in internal organs, primarily the liver (~90%) and lungs (~10%) [11,12]. The interconnected transmission cycle among animals, humans, and the environment underscores the importance of the One Health approach for effective control [13].

Sheep husbandry constitutes a primary economic pillar in these pastoral areas. Despite their importance, the epidemiology of *E. granulosus* infection in sheep remains poorly characterized, with existing studies being sporadic and geographically limited. Conducting continuous monitoring of *E. granulosus* in sheep provides insights into the control status of echinococcosis in China. In Xinjiang, Guo et al. [14,15] reported infection rates of 3.5% (44/1270) in northern Xinjiang and 4.5% (131/2898) in the Altai region, identifying two genotypes (G1 and G3). A higher prevalence was reported by Lan et al. [16], who found the prevalence of *E. granulosus* in sheep in Xinjiang to be as high as 17.1% (58/340), and two genotypes (G1 and G3) were detected. A comparative serosurvey by Zhang et al. [17] employing ELISA revealed a significantly higher seroprevalence in sheep from Qinghai Province than from Xinjiang. Similarly, an examination of cysts from slaughtered livestock by Su et al. [18] found that 56.2% (41/73) of *E. granulosus* cysts were from sheep and yaks, and genetic analysis detected three genotypes (sheep strains G1 and G3, and camel strain G6). Furthermore, Yang et al. [19] demonstrated an age-associated increase in prevalence in sheep and goats in western Sichuan Province, from 38% in 1–6-year-olds to 70% in 10–12-year-olds, with only the G1 genotype identified in the region.

However, comprehensive epidemiological data on *E. granulosus* infection in sheep within Yili Kazak Autonomous Prefecture (Yili Prefecture) remain scarce. Since CE infections in sheep are predominantly asymptomatic and reliable antemortem diagnostic techniques are lacking, direct postmortem inspection of the liver represents a more accurate method for determining the true prevalence of the disease. Therefore, the objectives of this study were twofold: (i) to conduct the first comprehensive assessment of *E. granulosus* prevalence in slaughterhouse-sampled sheep from nine counties across Yili Prefecture between 2020 and 2024 and (ii) to genetically characterize the circulating strains of *E. granulosus* using morphological and molecular analyses. Our findings provide critical baseline data to inform and refine future CE control strategies in this highly endemic region.

## 2. Materials and Methods

### 2.1. Study Area

This study was conducted in Yili Prefecture, located between 40°14′–49°10′ N and 80°09′–91°01′ E. The prefecture encompasses a total area of 56,500 km^2^, which includes 47.26 million acres of grassland and 9.67 million acres of forest land, making it the largest pastoral region in Xinjiang. According to official government statistics, the total mutton production from 2020 to 2024 was 370,700 tons, with an annual average of 74,140 tons. Production ranged from 55,300 tons in 2020 to a peak of 81,700 tons in 2023. From 2022 to 2024, the sheep inventory was 6.4 million, 5.9 million, and 5.2 million, respectively, while the number of sheep slaughtered was 4.3 million, 4.6 million, and 4.4 million, respectively (Supplementary Table S1).

### 2.2. Sample Collection and Visual Inspection

Sample collection was conducted uniformly across all counties in October each year. Inspections took place at slaughterhouses with a daily throughput exceeding 100 animals. Between fifty and eighty samples were inspected in each slaughterhouse, and the number of *E. granulosus* positive cases was recorded. Liver samples showing severe lesions-especially those with prominent surface cysts (including calcified cysts)-were transported to the laboratory for molecular testing.

During visual inspection, *E. granulosus* infection was diagnosed based on the presence of characteristic spherical cysts with thick, laminated, whitish walls and clear fluid or daughter cysts within. These features allow for a clear distinction from the biliary fibrosis and calcification caused by *Fasciola hepatica* and from the thin-walled, transparent vesicles attached to the hepatic surface caused by *Cysticercus tenuicollis*. Liver samples showing severe lesions (cysts larger than 5 cm) were transported to the laboratory for molecular testing.

**Figure 1:**
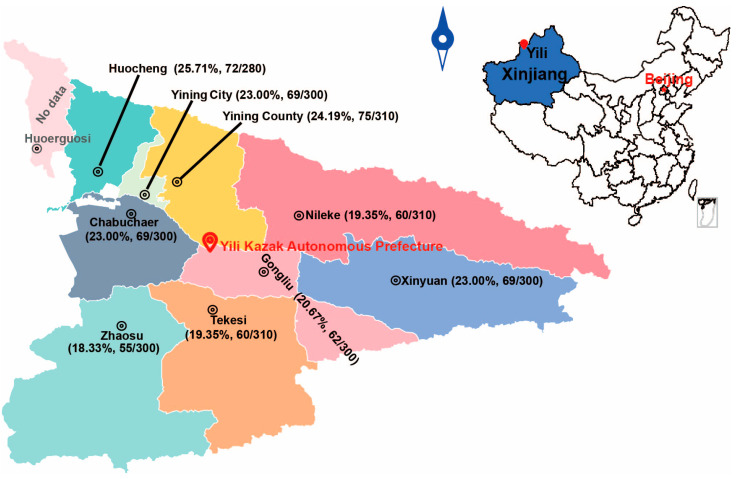
Map of sampling locations in the nine counties of Yili Prefecture. The numbers of total samples, positive samples, and infection rates are shown on the map.

From 2020 to 2024, a total of 2700 sheep livers were visually inspected for cystic lesions during slaughterhouse surveillance across 9 counties in Yili Prefecture of Xinjiang. The surveyed counties included Chabuchaer Xibo Autonomous County (Chabuchaer; *n* = 300), Gongliu County (*n* = 300), Huocheng County (*n* = 280), Nileke County (*n* = 310), Tekesi County (*n* = 300), Xinyuan County (*n* = 300), Yining City (*n* = 300), Yining County (*n* = 310), and Zhaosu County (*n* = 300) (Figure 1).

### 2.3. Morphological Characterization

Ninety severely infected cystic liver samples (including calcified cysts) were first washed with phosphate-buffered saline (PBS), and their surfaces were carefully wiped with 75% alcohol. Hydatid fluid was aseptically aspirated from the cysts using a 10 mL sterile syringe and then transferred to a 50 mL centrifuge tube for natural sedimentation. The protoscolices (PSCs) were pelleted via centrifugation at 500× *g* for 5 min, washed three times with PBS, and finally resuspended in 30 mL of PBS. For viability assessment, a 100 μL aliquot of the PSC suspension was stained with 1% methylene blue for 1 min and examined under a light microscope (Leica, Wetzlar, Germany). For molecular analysis, 1 mL aliquots of the PSC suspension were stored at −20 °C until DNA extraction.

### 2.4. DNA Extraction and PCR Amplification

The frozen PSC suspensions were thawed and homogenized using an orbital shaker (Jingxin Biotech, Shanghai, China). The samples were then centrifuged at 5000× *g* for 10 min, and the supernatant was carefully discarded. Genomic DNA was extracted using the TIANamp Genomic DNA Kit (TianGen Biotech, Beijing, China) according to the manufacturer’s instructions. The DNA was eluted in 100 μL of preheated (55 °C) elution buffer and stored at −20 °C until further analysis.

The NADH dehydrogenase subunit 2 (*nad2*) gene (~1500 bp) of *E. granulosus* was amplified using primers previously described by Guo et al. [20]: forward 5′-ATTTTGCGGTCGTCTCTGAT-3′ and reverse 5′-TCCACGAGACCAAGGATACC-3′. PCR was performed in a 25 μL reaction mixture containing 12.5 μL of 2× PCR buffer mix (Thermo Fisher Scientific, Waltham, MA, USA), 8.5 μL of nuclease-free water, 1 μL of each primer (10 μM), and 2 μL of template DNA. Amplification was carried out in a thermal cycler (Bio-Rad T100™, Hercules, CA, USA) under the following conditions: initial denaturation at 94 °C for 5 min; 35 cycles of denaturation at 94 °C for 30 s, annealing at 56 °C for 30 s, and extension at 72 °C for 2 min; and a final extension at 72 °C for 10 min. Each PCR run included both positive (genomic DNA from a confirmed *E. granulosus* sample) and negative (nuclease-free water instead of template DNA) controls.

### 2.5. Phylogenetic Analyses

PCR products were analyzed by electrophoresis on 1.0% agarose gels. Amplicons that yielded a band of the expected size were purified and sent for Sanger sequencing (Sunny Biotech, Shanghai, China). The obtained *nad2* sequences were compared to known sequences in the GenBank database using the BLAST algorithm (https://blast.ncbi.nlm.nih.gov/Blast.cgi) (accessed on 28 May 2025). Multiple sequence alignment was performed using Clustal W with default parameters, and the alignments were manually refined using MEGA X (version 10.2.6). A phylogenetic tree was reconstructed based on the aligned sequences using the neighbor-joining (NJ) method. All phylogenetic analyses employed the Kimura 2-parameter model, and the robustness of the inferred topology was assessed with 1000 bootstrap replicates.

A median-joining haplotype network was constructed using PopArt software (version 1.7) to visualize the relationships among mitochondrial DNA haplotypes (Haps). Genetic diversity indices, including the number of Haps, haplotype diversity (Hd), and nucleotide diversity (π), were calculated using DnaSP software (v6). Neutrality tests, including Tajima’s *D* and Fu’s *Fs*, were performed to detect deviations from population equilibrium. Population genetic structure was evaluated by calculating pairwise fixation indices (F_st_) in Arlequin software (version 3.5.2.2). All unique *nad2* sequences generated in this study were deposited in the GenBank database under accession numbers PX430486 to PX430498.

### 2.6. Statistical Analysis

The prevalence of *E. granulosus* infection in sheep livers, expressed as a percentage with 95% confidence intervals (CIs), was calculated for each of the nine study counties. Differences in prevalence across counties and years were assessed using Pearson’s chi-square test (*χ*^2^), while year and county as risk factors were assessed by multivariable logistic regression. All statistical analyses were performed using IBM SPSS Statistics (version 22.0; IBM Corp., Armonk, NY, USA). The statistical analyses for Tajima’s *D*, Fu’s *Fs*, and F_st_ values across different counties were performed automatically by the respective software modules. Specifically, Tajima’s *D* and Fu’s *Fs* were evaluated using repeated data simulations, while the F_st_ values were assessed via a permutation test. A *p*-value less than 0.05 was considered statistically significant for all analyses.

## 3. Results

### 3.1. Morphological Characteristics

**Figure 2:**
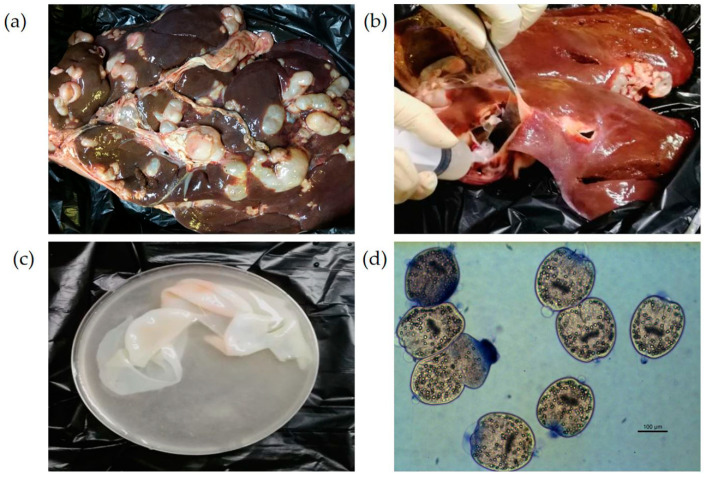
The detection of sheep hydatidosis with the naked eye with the pathology and morphology characteristics: (**a**) cyst characteristics, embedded on the surface of the sheep liver; (**b**) liver with cavities caused by *E. granulosus* cysts; (**c**) an internal cyst that could be dragged out from *E. granulosus* cysts, which was full of a large amount of hydatid fluid and PSCs; (**d**) PSCs (200×) stained with methylene blue.

Hydatid cysts, the larval stage of *E. granulosus*, were predominantly observed in the livers of infected sheep. The cysts, varying in size, were embedded on the hepatic surface. Upon dissection, these cysts were found to contain turbid hydatid fluid and numerous PSCs, which settled as a white sediment. The PSCs exhibited elliptical or gourd-like morphologies, with an average diameter ranging from 70 to 80 μm. When stained with 1% methylene blue, the PSCs took up the dye slightly. The characteristic hooks were observed in the center of the elliptical PSCs and at the rostellar end of the gourd-shaped forms (Figure 2).

### 3.2. Overall Prevalence of E. granulosus in Sheep Across Different Districts

**Figure 3:**
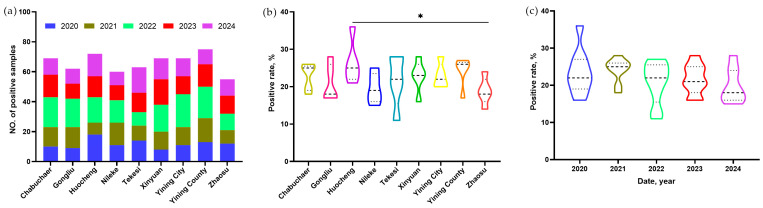
Prevalence of sheep CE across nine counties in Yili Prefecture (2020–2024): (**a**) number of positive samples per region (color-coded by year); (**b**) violin plots showing positive rates in different regions, where * indicates a significant difference (*p* < 0.05); (**c**) violin plots showing annual prevalence across the study period. The middle dashed line in the figures represents the median, which is the value in the middle position after sorting all the data from smallest to largest.

A five-year (2020–2024) longitudinal surveillance study was conducted to investigate the prevalence of *E. granulosus* in sheep across nine counties of Yili Prefecture. Visual inspection of 2700 sheep livers identified 594 positive cases, resulting in an overall infection prevalence of 22.0% (594/2700) (Table 1). The county-level prevalence ranged from 18.3% to 25.7%. The highest prevalence was recorded in Huocheng County (25.7%, 72/280), followed by Yining County (24.2%, 75/310). Chabuchaer County, Xinyuan County, and Yining City exhibited a similar prevalence of 23.0% (69/300 each). Lower prevalence rates were observed in Tekesi County (21.0%, 63/300), Gongliu County (20.7%, 62/300), and Nileke County (19.4%, 60/310). The lowest prevalence was found in Zhaosu County (18.3%, 55/300). Statistical analysis revealed a significant difference in prevalence between Huocheng County and Zhaosu County (*p* < 0.05), while no significant differences were observed among the other counties (*p* > 0.05) (Table 1, Figure 3a,b).

### 3.3. Temporal Variation in E. granulosus Prevalence in Sheep from 2020 to 2024

Annual surveillance data from 2020 to 2024 revealed fluctuations in prevalence rates, although these changes were not statistically significant. The highest prevalence was observed in 2021 (24.2%, 195/806), followed by 2020 (23.5%, 106/450), 2023 (21.9%, 118/540), and 2022 (21.1%, 152/720). The lowest prevalence was recorded in 2024 (20.2%, 109/540). Statistical analysis confirmed that the interannual variations in prevalence were not significant (*p* > 0.05) (Table 2, Figure 3c).

### 3.4. Temporal Variation in E. granulosus Prevalence Within Individual Counties

Analysis of year-to-year fluctuations within each county revealed considerable heterogeneity in prevalence trends. The prevalence remained above 20% across all five years in Huocheng County (range: 21.2–36.0%) and Yining City (range: 20.0–27.5%). In contrast, Gongliu, Nileke, and Zhaosu counties recorded prevalence rates below 20% in three out of the five study years. Isolated instances of annual prevalence dropping below 20% were observed in Chabuchaer (18.3% in 2024), Tekesi (11.3% in 2022), Xinyuan (16.0% in 2020), and Yining County (16.7% in 2024). Statistical analysis indicated that significant interannual variation (*p* < 0.05) occurred only in Tekesi County; no significant temporal fluctuations were detected in the other eight counties (*p* > 0.05) (Table 3, Figure 3a).

### 3.5. Spatial Variation in E. granulosus Prevalence Within Individual Years

The spatial distribution of prevalence across the nine counties was analyzed for each year. The widest range of prevalence was observed in 2020 (from 16.0% in Xinyuan County to 36.0% in Huocheng County) and in 2022 (from 11.3% in Tekesi County to 27.5% in Yining City). In 2021, the prevalence exceeded 20% in all counties (range: 20.0–28.0%) except Zhaosu County (18.0%, 9/50). In 2023, Gongliu and Nileke counties shared an identical prevalence of 16.7% (10/60), while prevalence in the remaining seven counties was above 20% (range: 20.0–28.3%). In 2024, four counties exhibited a prevalence above 20%: Huocheng (25.0%, 15/60), Tekesi (28.3%, 17/60), Xinyuan (23.3%, 14/60), and Yining City (20.0%, 12/60). The prevalence in the other five counties was below 20% (range: 15.0–18.3%). Significant differences in prevalence among counties were detected in 2020 and 2022 (*p* < 0.05), but not in other years (Table 3).

### 3.6. Multivariate Logistic Regression Analysis of Risk Factors for E. granulosus Prevalence

Multivariable logistic regression analysis showed that Huocheng County had the highest infection risk (OR = 1.56), which was significantly different from Zhaosu County (the reference group, which had the lowest infection rate). In contrast, no statistically significant differences were observed between the other counties and Zhaosu County (*p* > 0.05; Table 4). Additionally, using the first year (2020) as the reference, no significant differences in infection risk were found across the study years (*p* > 0.05; Table 5). Both the spatial and temporal results were consistent with the chi-square analysis.

### 3.7. Molecular Identification and Phylogenetic Relationships

Following macroscopic inspection, 90 severely infected liver samples were selected for molecular analysis. PCR amplification of the *nad2* gene was successful for 83 samples (92.2% success rate). Sequencing and subsequent genotyping identified 79 samples (95.2%) as *E. granulosus* sensu stricto (s. s.) genotype G1 and 4 samples (4.8%) as genotype G3 (Table 6). The G1 genotype was ubiquitous, detected in all nine counties. The G3 genotype was found in four counties: Gongliu, Nileke, Tekesi, and Yining (Table 6).

**Figure 4:**
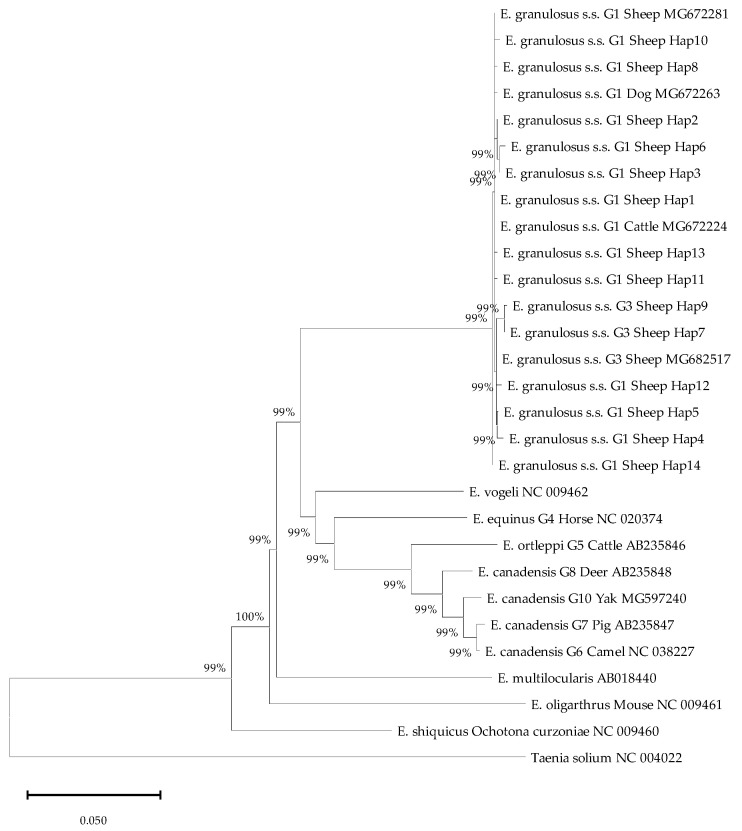
Phylogenetic relationships among *Echinococcus* spp. inferred via neighbor-joining analysis using the NADH dehydrogenase subunit 2 (*nad2*) gene.

Analysis of the 83 *nad2* sequences defined 14 distinct haplotypes (Hap1–Hap14). Hap1 was the dominant haplotype, identified in all nine counties and comprising 47.0% (39/83) of all sequences. Hap2 was the second most prevalent, detected in eight counties and accounting for 21.7% (18/83) of sequences (Table 7). The distribution of haplotype diversity varied by county. Yining City harbored the greatest number of haplotypes (*n* = 7), followed by Nileke County (*n* = 6). In contrast, Huocheng and Xinyuan counties contained the fewest, with only three haplotypes each (Table 8). Phylogenetic reconstruction confirmed that Hap7 and Hap9 clustered within the G3 genotype, while other Haps were associated with the G1 genotype (Figure 4).

### 3.8. Population Genetic Diversity of E. granulosus *s.s.* Across Counties

Genetic diversity indices for the *nad2* gene of *E. granulosus* s. s. were calculated for each county using DnaSP v6 (Table 9). Overall, Hd ranged from 0.607 to 0.911, and π ranged from 0.00143 to 0.00284. Yining City exhibited the highest genetic diversity, with the maximum Hd value (0.911) and a high π value (0.00277). In contrast, Huocheng County displayed the lowest genetic diversity, with both the minimum Hd (0.607) and π (0.00143). Nileke County also demonstrated high diversity, possessing the highest π value (0.00284) and a high Hd (0.833). Conversely, Xinyuan County showed a relatively low Hd (0.722) coupled with a moderate π value (0.00228).

Neutrality tests yielded mostly negative values for both Tajima’s *D* and Fu’s *Fs* across most counties, though none were statistically significant (*p* > 0.05). Tajima’s *D* was negative in eight counties, with a positive value observed only in Xinyuan (Tajima’s *D* = 0.497). Similarly, Fu’s *Fs* was negative in seven counties, with Yining City showing the most strongly negative value (Fu’s *Fs* = −2.590). Huocheng and Xinyuan counties were exceptions, showing positive Fu’s *Fs* values (0.671 and 1.855, respectively) (Table 9).

Pairwise fixation index (F_st_) analysis revealed a lack of significant genetic differentiation among any of the county subpopulations (*p* > 0.05). All F_st_ values were negligible, ranging from −0.0996 (between Nileke and Yining) to 0.0110 (between Huocheng and Yining), indicating high gene flow and a panmictic population structure (Table 10).

### 3.9. Haplotype Network Analysis

**Figure 5:**
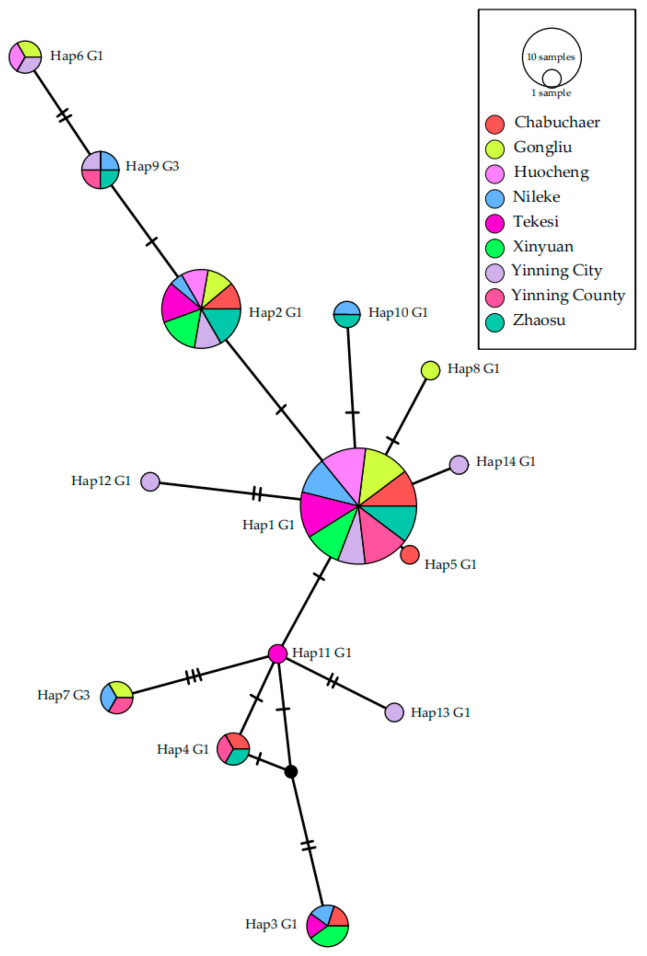
Haplotype networks were generated from the *nad2* gene of *E. granulosus.* A circular icon represents Haps containing the same sequence, and the circle size is proportional to the number of individuals showing such specific Haps in this study. Black dot indicate potential haplotype that was not identified in this study. Slashes represent SNP sites, with the number of slashes corresponding to the possible number of SNP sites at that position (one slash for one site, two slashes for two sites, and so on).

A median-joining network was reconstructed to elucidate the genealogical relationships among the 14 identified *E. granulosus* haplotypes (Figure 5). The network topology revealed a star-like structure, with the most abundant haplotype, Hap1, occupying a central position. Hap1 was directly connected to seven other haplotypes (Hap2, Hap5, Hap8, Hap10, Hap11, Hap12, and Hap14), suggesting their recent derivation from this common ancestor. Hap11 functioned as a secondary hub, linking Hap1 to four additional haplotypes (Hap3, Hap4, Hap7, and Hap13). The other dominant haplotype, Hap2, exhibited a simpler connection pattern, linked only to Hap1 and Hap9. Hap6 was the most peripherally located haplotype, separated from the central Hap1 by multiple mutational steps, with Hap9 and Hap2 acting as intermediate nodes. The presence of inferred, unsampled (missing) haplotypes was suggested by the reticulate connections, particularly those forming triangular loops between Hap4, Hap11, and an unnamed node (indicated by a black dot in Figure 5).

## 4. Discussion

The Yili River Valley is a pivotal sheep breeding base in Xinjiang and a nationally significant livestock hub, possessing exceptional natural conditions for pastoralism. Despite this, the region has consistently documented persistently high rates of echinococcosis. This endemicity is likely driven by a confluence of factors, including a harsh climate, extensive pastoral systems, suboptimal veterinary public health infrastructure, and frequent cross-border livestock movements. Previous localized studies have reported *E. granulosus* infection rates in sheep that exceed the national average by two to six-fold [16,21]. Nonetheless, comprehensive, longitudinal surveillance data at a prefecture-wide scale have been lacking. This knowledge gap has obscured the dynamic epidemiological trends of CE and hindered an accurate assessment of transnational transmission risks, underscoring the critical need for the present study.

Our findings address a significant gap in the epidemiological surveillance of *E. granulosus* in sheep. While many previous studies have focused primarily on genotyping and genetic polymorphism using PCR-confirmed samples, our approach integrated large-scale, longitudinal monitoring with macroscopic examination. Through a five-year surveillance program across nine counties in Yili Prefecture, we documented a consistently high overall prevalence of 22.0% (95% CI: 20.5–23.6). This prevalence is markedly higher than infection rates previously reported in northern Xinjiang. For instance, it substantially exceeds the 3.9% (14/361) reported in Yili Prefecture and 4.5% (131/2898) in the Altai Region by Guo et al. [14,15], as well as the 2.3% (390/17,215) documented in Emin County of Tacheng Region by Yang et al. [22]. The higher prevalence observed in our study is likely attributable to the inclusion of early and mild infections that were detected macroscopically but are often missed by PCR. This is supported by our practical experience: when we attempted molecular genotyping on a subset of our macroscopically positive samples, PCR amplification was consistently unsuccessful in samples with minimal pathological changes, suggesting a low parasitic DNA load below the assay’s detection limit.

Previous studies have indicated that the infection rate of *E. granulosus* in sheep is generally higher in pastoral areas than in agricultural regions of Xinjiang [23]. However, our investigation in the Yili Prefecture revealed no significant difference in sheep infection rates across its various counties (the total infection rates fluctuating from 18.3% to 25.7%). This homogeneity may be attributed to the prefecture’s distinctive topography and mixed farming–pastoral landscape. Located in northwestern Xinjiang and bordering Kazakhstan, Yili Prefecture is surrounded by mountains on three sides, with the Yili River Valley serving as its core geographical unit [24]. Agricultural and pastoral areas are interwoven and indistinctly demarcated throughout the prefecture, often exhibiting a mixed “agro-pastoral” pattern, which may explain the relatively uniform spatial distribution of *E. granulosus* infections. On the other hand, significant year-to-year variations were observed in some areas, with prevalence rates ranging from, for example, 11.3% in Tekesi County (2022) to 36.0% in Huocheng County (2020). A potential explanation for this disparity lies in the heterogeneous origins of the sheep, given that control strategies likely vary from one pasture to another. On the contrary, five years of surveillance data from 2020 to 2024 revealed no significant differences in annual prevalence rates (*p* > 0.05). This may be attributed to the relative stability of the annual CE control strategies in the Yili region, as the prevention and control measures were strictly implemented in accordance with the annual guidelines issued by the Department of Agriculture and Rural Affairs of Xinjiang, primarily consisting of dog deworming and lamb vaccination. However, this study primarily focused on adult sheep (mostly aged 1–4 years) from slaughterhouses, in which the protective immunity from earlier vaccination may have waned or diminished. Furthermore, the vast grasslands in the Yili region are frequented by wildlife such as foxes and wolves, which serve as significant reservoirs of infection. Relying solely on deworming domestic dogs may therefore be insufficient to disrupt the transmission cycle at its source. The persistently high prevalence can seriously affect sheep health, leading to issues such as sheep death, slow growth, prolonged time to slaughter, organ discards and increased rearing costs [25]. Given that multiple factors, such as livestock farming scale and annual climatic variations, can influence the transmission of CE, it is necessary to develop control strategies through multi-sectoral collaboration [26,27].

Furthermore, a notably higher infection rate was observed in Yining City (23.0%), and the highest genetic diversity (Hd = 0.911, π = 0.00277) was also found in this city, likely associated with its unique geographical and socioeconomic role. As the capital and most populous city of Yili Prefecture, Yining City has the highest daily sheep slaughter volume in the region. Although it is not a major agricultural or pastoral production area itself, most of the sheep slaughtered in the city are sourced from various counties across Yili Prefecture. This influx of animals from multiple origins may contribute to the higher observed infection rate and genetic diversity in Yining City.

Molecular genotyping of hydatid cysts, targeting the *nad2* gene, confirmed the exclusive circulation of the *E. granulosus* s. s. G1 genotype in our samples. This finding is consistent with the global predominance of this livestock-adapted strain. The dominance of G1 aligns with recent reports from the East Tianshan Mountains in Xinjiang [28] and is well-documented across other endemic provinces in China, including the Altai region of northern Xinjiang [15], Xizang Autonomous Region [18,29,30,31], Ningxia Hui Autonomous Region [32], and Sichuan Province [19]. Globally, the G1 genotype is notorious for its pronounced zoonotic potential, constituting a major public health threat from the Mediterranean Basin [33] to Pakistan [34], Kenya [35], and North Africa [36]. The shared global genotype profile suggests the possibility of cross-border transmission pathways. However, validating the direction and intensity of such transmission requires future high-resolution population genetic studies (e.g., using microsatellite or SNP-based haplotyping). Furthermore, as this study primarily analyzed samples with more severe lesions (including calcified cysts), the findings may be subject to selection bias. Future studies should therefore include and successfully analyze samples with mild lesions to comprehensively validate the present results.

The dominance of the G1 genotype in sheep reservoirs directly amplifies the human health risk within a One Health framework. This is critically relevant to Yili, as human CE cases in Xinjiang are mainly caused by the G1 genotype [23,37], and the prevalence of *Echinococcus* in dogs can reach 5.8% (5557/200,165) in Xinjiang [38]. These factors sustain a pernicious transmission cycle involving dogs (definitive hosts), the environment (eggs), and both sheep and humans (intermediate hosts) [39]. This cycle is profoundly amplified by traditional slaughter practices, where infected offal is routinely fed to dogs. This dangerous feedback loop mirrors transmission dynamics observed in other endemic regions such as Kenya and Pakistan [35,40,41], highlighting a universal challenge in CE control [42]. However, this study did not examine environmental or canine fecal samples. Therefore, it remains unclear whether the persistently high prevalence of ovine CE in Yili is associated with dogs. Future studies are needed to investigate *E. granulosus* infections in dogs in Yili.

Our study identified 14 Haps of *E. granulosus* s.s. circulating in sheep across Yili Prefecture. This level of haplotype diversity is broadly consistent with several previous reports from Xinjiang and other endemic regions in China. For instance, studies targeting the *cox1* gene identified 12 Haps in Hejing County [16] and 16 Haps in the Altai region [15], while 13 Haps were reported in sheep from Xizang [43]. In contrast, other studies have reported lower haplotype diversity within Xinjiang. Guo et al. [14] found only seven Haps across a wide geographical area encompassing Urumqi, Yili, Tacheng, and Altai. Similarly, Yan et al. [44] documented 10 Haps in Yili Prefecture and 9 Haps in Changji Hui Autonomous Prefecture of Xinjiang. The discrepancies in reported haplotype diversity across studies are likely multifactorial. Key influencing variables include the sample size (with larger, more comprehensive surveys like ours capturing greater diversity), the geographical scale and sampling strategy (targeting a single prefecture vs. multiple pooled regions), the choice of genetic marker (*nad2* in our study vs. cox1 in others), and potential temporal fluctuations in parasite population structure.

The F_st_ values, which were all negligible and not significantly different from zero (range: −0.09964 to 0.01099; *p* > 0.05), indicate a pronounced lack of genetic differentiation among *E. granulosus* populations across the nine counties of Yili Prefecture. Notably, the lowest F_st_ value was observed between Nileke and Yining County (F_st_ = −0.09964), suggesting particularly frequent genetic exchange between these two populations. Actually, the two counties are adjacent (Figure 1), which is likely the reason for the minimal genetic variation in *E. granulosus* between them. Similarly, pairwise comparisons involving the central hub of Yining City (e.g., with Xinyuan and Yining County) and other counties (e.g., Xinyuan with Zhaosu) yielded values asymptotically approaching zero, further reinforcing the genetic homogeneity of the parasite population throughout the region. This panmixia is most parsimoniously explained by the extensive movement of infected intermediate hosts (sheep) through regional livestock trade and pastoral migration patterns.

This study has several limitations that should be considered when interpreting the results. First, the reliance on animals presented for slaughter means the sample may not be fully representative of the general sheep population in the region, as factors such as age, health, and management practices of slaughtered animals could introduce a potential selection bias. Second, the absence of age and sex-stratified data prevented the analysis of associated risk factors, which is crucial for understanding transmission dynamics. For future research, serological surveys in farms, combined with environmental sampling and the testing of feces from definitive host dogs, are recommended to enable a comprehensive assessment of CE in Yili, Xinjiang.

## 5. Conclusions

This five-year, comprehensive surveillance study establishes a persistently high burden (22.0%) of *E. granulosus* infection in sheep within China’s key pastoral region of Yili Prefecture, identifying Huocheng County and Yining City as particular hyperendemic foci. Molecular characterization revealed a striking genetic homogeneity, with the zoonotically significant G1 genotype (*E. granulosus* s. s.) accounting for 95.2% of infections. Population genetic analysis identified 14 haplotypes, with the dominant and centrally located Hap1 indicative of a recently expanded and interconnected parasite population. Therefore, the dominance of the G1 genotype, coupled with the high infection pressure in sheep reservoirs, indicates a substantial and ongoing zoonotic risk to human populations in the region. Collectively, our findings underscore the entrenched nature of CE transmission in northwestern China. These findings highlight the endemicity of echinococcosis in the region and provide critical genetic insights for guiding future control strategies.

## Figures and Tables

**Table 1:** The total infection rates of *E. granulosus* in sheep from different counties.

County	No. Sample	No. Positive	Positive Rate (%)	95% CI
Chabuchaer	300	69	23.0	18.4–28.2
Gongliu	300	62	20.7	16.2–25.7
Huocheng	280	72	25.7	20.7–31.3
Nileke	310	60	19.4	15.1–24.2
Tekesi	300	63	21.0	16.5–26.1
Xinyuan	300	69	23.0	18.4–28.2
Yining City	300	69	23.0	18.4–28.2
Yining County	310	75	24.2	19.5–29.4
Zhaosu	300	55	18.3%	14.1–23.2
Total	2700	594	22.0%	20.5–23.6

**Table 2:** The total infection rates of *E. granulosus* in sheep from different years.

Year	No. Sample	No. Positive	Positive Rate (%)	95% CI
2020	450	106	23.6	19.7–27.8
2021	450	109	24.2	20.3–28.5
2022	720	152	21.1	18.2–24.3
2023	540	118	21.9	18.4–25.6
2024	540	109	20.2	16.9–23.8
Total	2700	594	22.0%	20.5–23.6

**Table 3:** The infection rates of *E. granulosus* in sheep across 9 counties from 2020 to 2024.

County	Year [Positive Rate (%), No. Positive/No. Sample]				
	2020	2021	2022	2023	2024
Chabuchaer	20.0%, 10/50	26.0%, 13/50	25.0%, 20/80	25.0%, 15/60	18.3%, 11/60
Gongliu	18.0%, 9/50	28.0%, 14/50	23.8%, 19/80	16.7%, 10/60	16.7%, 10/60
Huocheng	36.0%, 18/50	26.7%, 8/30	21.2%, 17/80	23.3%, 14/60	25.0%, 15/60
Nileke	22.0%, 11/50	25.0%, 15/60	18.8%, 15/80	16.7%, 10/60	15.0%, 9/60
Tekesi	28.0%, 14/50	20.0%, 10/50	11.3%, 9/80	21.7%, 13/60	28.3%, 17/60
Xinyuan	16.0%, 8/50	24.0%, 12/50	22.5%, 18/80	28.3%, 17/60	23.3%, 14/60
Yining City	22.0%, 11/50	24.0%, 12/50	27.5%, 22/80	20.0%, 12/60	20.0%, 12/60
Yining County	26.0%, 13/50	26.7%, 16/60	26.3%, 21/80	25.0%, 15/60	16.7%, 10/60
Zhaosu	24.0%, 12/50	18.0%, 9/50	13.8%, 11/80	20.0%, 12/60	18.3%, 11/60

**Table 4:** Multivariate logistic regression analysis of *E. granulosus* infection by county.

County	OR	95% CI	*p*-Value
Zhaosu	1.00	/	/
Chabuchaer	1.33	0.89–1.98	0.16
Gongliu	1.16	0.77–1.74	0.47
Huocheng	1.56	1.05–2.32	0.03
Nileke	1.06	0.71–1.60	0.76
Tekesi	1.19	0.79–1.77	0.41
Xinyuan	1.33	0.89–1.98	0.16
Yining City	1.33	0.89–1.98	0.16
Yining County	1.42	0.96–2.09	0.08

**Table 5:** Multivariate logistic regression analysis of *E. granulosus* infection by year.

Year	OR	95% CI	*p*-Value
2020	1.00	/	/
2021	1.05	0.77–1.43	0.76
2022	0.90	0.68–1.19	0.45
2023	0.86	0.64–1.16	0.32
2024	0.80	0.59–1.09	0.15

**Table 6:** The genotypes of *E. granulosus* in sheep across different counties.

County	Molecular Test Number	Successfully Amplified Number	*E. granulosus* Genotypes (%)	
			G1	G3
Chabuchaer	10	9	9 (100.0)	0
Gongliu	10	10	9 (90.0)	1 (10.0)
Huocheng	10	8	8 (100.0)	0
Nileke	10	9	8 (88.9)	1 (11.1)
Tekesi	10	10	9 (90.0)	1 (10.0)
Xinyuan	10	9	9 (100.0)	0
Yining City	10	10	10 (100.0)	0
Yining County	10	8	7 (87.5)	1 (12.5)
Zhaosu	10	10	10 (100.0)	0
Total	90	83	79 (95.2)	4 (4.8)

**Table 7:** Geographical distribution of the *E. granulosus* haplotypes.

Haplotype	Genotype	Number	Geographical Distribution (No.)
Hap1	G1	39	Chabuchaer (4), Gongliu (5), Huocheng (5), Nileke (4), Tekesi (5), Xinyuan (4), Yining City (3), Yining County (5), Zhaosu (4)
Hap2	G1	18	Chabuchaer (2), Gongliu (2), Huocheng (2), Nileke (1), Tekesi (3), Xinyuan (3), Yining City (2), Zhaosu (3)
Hap3	G1	5	Chabuchaer (1), Nileke (1), Tekesi (1), Xinyuan (2)
Hap4	G1	4	Nileke (1), Yining City (1), Yining County (1), Zhaosu (1)
Hap5	G1	3	Chabuchaer (1), Yining County (1), Zhaosu (1)
Hap6	G1	3	Gongliu (1), Huocheng (1), Yining City (1)
Hap7	G3	3	Gongliu (1), Nileke (1), Yining County (1)
Hap8	G1	2	Nileke (1), Zhaosu (1)
Hap9	G3	1	Tekesi (1)
Hap10	G1	1	Yining City (1)
Hap11	G1	1	Gongliu (1)
Hap12	G1	1	Yining City (1)
Hap13	G1	1	Yining City (1)
Hap14	G1	1	Chabuchaer (1)

**Table 8:** The distribution of *E. granulosus* haplotypes in different counties.

County	Haplotype Types (No.)	Haplotypes Distribution (No.)
Chabuchaer	5	Hap1 (4), Hap2 (2), Hap3 (1), Hap5 (1), Hap14 (1)
Gongliu	5	Hap1 (5), Hap2 (2), Hap6 (1), Hap7 (1), Hap11 (1)
Huocheng	3	Hap1 (5), Hap2 (2), Hap6 (1)
Nileke	6	Hap1 (4), Hap2 (1), Hap3 (1), Hap4 (1), Hap7 (1), Hap8 (1)
Tekesi	4	Hap1 (5), Hap2 (3), Hap3 (1), Hap9 (1)
Xinyuan	3	Hap1 (4), Hap2 (3), Hap3 (2)
Yining City	7	Hap1 (3), Hap2 (2), Hap4 (1), Hap6 (1), Hap10 (1), Hap12 (1), Hap13 (1)
Yining County	4	Hap1 (5), Hap4 (1), Hap5 (1), Hap7 (1)
Zhaosu	5	Hap1 (4), Hap2 (3), Hap4 (1), Hap5 (1), Hap8 (1)
Total	14	Hap1 (39), Hap2 (18), Hap3 (5), Hap4 (4), Hap5 (3), Hap6 (3), Hap7 (3), Hap8 (2), Hap9 (1), Hap10 (1), Hap11 (1), Hap12 (1), Hap13 (1), Hap14 (1)

**Table 9:** Diversity and neutrality indices for different populations of *E. granulosus* subpopulations from different counties of Yili Prefecture.

County	n ^1^	h ^2^	Hd ^3^	π ^4^	Tajima’s *D*	Statistical Significance	Fu’s *Fs*
Chabuchaer	9	5	0.7397	0.103395	−0.68479	*p* = 0.254	−1.00932
Gongliu	10	5	0.7556	0.114815	−1.53425	*p* = 0.068	−0.52754
Huocheng	8	3	0.6071	0.071429	−0.72673	*p* = 0.261	0.67071
Nileke	9	6	0.8333	0.141975	−1.42187	*p* = 0.081	−1.54903
Tekesi	10	4	0.7111	0.079012	−0.78318	*p* = 0.245	−0.14158
Xinyuan	9	3	0.7222	0.114198	0.49704	*p* = 0.712	1.85521
Yining City	10	7	0.9111	0.138272	−1.31156	*p* = 0.096	−2.58952
Yining County	8	4	0.6429	0.107143	−1.35929	*p* = 0.101	0.08149
Zhaosu	10	5	0.8000	0.074074	−0.98485	*p* = 0.209	−1.54678

^1^ Number of isolates examined; ^2^ number of Haps; ^3^ haplotype diversity; ^4^ nucleotide diversity.

**Table 10:** F_ST_ Values of *nad2* gene of *E. granulosus* from different counties of Yili Prefecture.

County	Chabuchaer	Gongliu	Huocheng	Nileke	Tekesi	Xinyuan	Yining City	Yining County	Zhaosu
Chabuchaer	0.00000								
Gongliu	−0.03418	0.00000							
Huocheng	−0.01408	−0.08597	0.00000						
Nileke	−0.07756	−0.07679	−0.03639	0.00000					
Tekesi	−0.09337	−0.05087	−0.04269	−0.07579	0.00000				
Xinyuan	−0.08647	−0.01369	−0.00573	−0.06714	−0.08527	0.00000			
Yining City	−0.01066	−0.05453	−0.08472	−0.03168	−0.02924	−0.00356	0.00000		
Yining County	−0.05736	−0.06614	0.01099	−0.09964	−0.03538	0.00349	−0.00726	0.00000	
Zhaosu	−0.03242	−0.04938	−0.06938	−0.04472	−0.05174	−0.00078	−0.05006	−0.01467	0.00000

## Data Availability

The original contributions presented in this study are included in the article/Supplementary Material. Further inquiries can be directed to the corresponding authors.

## Supplementary Materials

The following supporting information can be downloaded at: https://www.mdpi.com/article/10.3390/pathogens15010040/s1, Table S1: Statistics on mutton production and sheep numbers in Yili Prefecture from 2020 to 2024.

## References

[1] Manterola C., Totomoch-Serra A., Rojas C., Riffo-Campos A.L., Garcia-Mendez N. (2022). *Echinococcus granulosus sensu lato* genotypes in different hosts worldwide: A systematic review. Acta Parasitol..

[2] Budke C.M., Deplazes P., Torgerson P.R. (2006). Global socioeconomic impact of cystic echinococcosis. Emerg. Infect. Dis..

[3] Massolo A., Simoncini A., Romig T. (2022). The ‘bridge effect’ by intermediate hosts may explain differential distributions of *Echinococcus* species. Trends Parasitol..

[4] Shams M., Khazaei S., Naserifar R., Shariatzadeh S.A., Anvari D., Montazeri F., Pirestani M., Majidiani H. (2022). Global distribution of *Echinococcus granulosus* genotypes in domestic and wild canids: A systematic review and meta-analysis. Parasitology.

[5] Wen H., Vuitton L., Tuxun T., Li J., Vuitton D.A., Zhang W., McManus D.P. (2019). Echinococcosis: Advances in the 21st Century. Clin. Microbiol. Rev..

[6] Eckert J., Deplazes P. (2004). Biological, epidemiological, and clinical aspects of echinococcosis, a zoonosis of increasing concern. Clin. Microbiol. Rev..

[7] Fu M., Han S., Xue C., Wang X., Liu B., Wang Y., Wang L., Wei S., Cui X., Zhang T. (2020). Contribution to the echinococcosis control programme in China by NIPD-CTDR. Adv. Parasitol..

[8] Wang X., Kui Y., Xue C., Wang Q., Zheng C., Zhao J., Yang Y., Jiang X., Gong-Sang Q., Ma X. (2025). Past, present and future epidemiology of echinococcosis in China based on nationwide surveillance data 2004-2022. J. Infect..

[9] Gu H., Hu Y., Guo S., Jin Y., Chen W., Huang C., Hu Z., Li F., Liu J. (2024). China’s prevention and control experience of echinococcosis: A 19-year retrospective. J. Helminthol..

[10] Li K., Shahzad M. (2019). Epidemiology of cystic echinococcosis in China (2004–2016). Travel Med. Infect. Dis..

[11] Craig P.S., Hegglin D., Lightowlers M.W., Torgerson P.R., Wang Q. (2017). Echinococcosis: Control and prevention. Adv. Parasit..

[12] Larrieu E.J., Frider B. (2001). Human cystic echinococcosis: Contributions to the natural history of the disease. Ann. Trop. Med. Parasitol..

[13] Tian T., Miao L., Wang W., Zhou X. (2024). Global, regional and national burden of human cystic echinococcosis from 1990 to 2019: A systematic analysis for the global burden of disease study 2019. Trop. Med. Infect. Dis..

[14] Guo B., Zhang Z., Zheng X., Guo Y., Guo G., Zhao L., Cai R., Wang B., Yang M., Shou X. (2019). Prevalence and molecular characterization of *Echinococcus granulosus sensu stricto* in Northern Xinjiang, China. Korean J. Parasitol..

[15] Guo B., Zhao L., Zhao L., Mi R., Zhang X., Wang B., Guo G., Ren Y., Qi W., Zhang Z. (2023). Survey and molecular characterization of *Echinococcus granulosus sensu stricto* from livestock and humans in the Altai Region of Xinjiang, China. Pathogens.

[16] Lan Q., Bianba Z., Mo X., Zheng G., Bold B., He G., Gao H., Hu W., Zhang T., Zhou X. (2025). *Echinococcus* infection and metacestode fertility in yaks and sheep-four provincial-level administrative divisions, Northwestern China, 2023. China CDC Weekly.

[17] Zhang X., Wei C., Lv Y., Mi R., Guo B., Rahman S.U., Zhang Y., Cheng L., Jia H., Huang Y. (2023). EgSeverin and Eg14-3-3zeta from *Echinococcus granulosus* are potential antigens for serological diagnosis of echinococcosis in dogs and sheep. Microb. Pathogenesis.

[18] Su Z., Wang D., Sizhu S., Luo R., Wang Q., Shi B., Tang W. (2024). Study on the genotypes of *Echinococcus granulosus* in yaks and sheep from Langkazi County in Tibet Autonomous Region of China based on mitochondrial *cox1* and *nad1*. Parasitol. Res..

[19] Yang Y.R., McManus D.P., Huang Y., Heath D.D. (2009). *Echinococcus granulosus* infection and options for control of cystic echinococcosis in Tibetan communities of Western Sichuan Province, China. PLoS Negl. Trop. Dis..

[20] Guo B., Cairen, Zhao L., Aimulajiang K., Tang W., Wu C., Yimingjiang M., Wu J., Mi R., Wen H. (2024). First report of *Echinococcus granulosus* genotype 1 in a wild boar (*Sus scrofa*) from China. Parasitol. Res..

[21] Wang Z., Wang X., Liu X. (2008). Echinococcosis in China, a review of the epidemiology of *Echinococcus* spp.. Ecohealth.

[22] Yang S., Wu W., Tian T., Zhao J., Chen K., Wang Q., Feng Z. (2015). Prevalence of cystic echinococcosis in slaughtered sheep as an indicator to assess control progress in Emin County, Xinjiang, China. Korean J. Parasitol..

[23] Meng Q., Qiao J. (2023). Epidemiology and control strategies of hydatid disease in Xinjiang. Prog. Vet. Med..

[24] Huang Q., Zhao Y., He Q. (2015). The daily variation characteristics of summer precipitation over the Yili River Valley, Xinjiang. J. Glaciol. Geocryol..

[25] Ma J., Liu X., He Z., Zhang H., Ma Y., Fan Z., Zhao X., Zhi J., Cao Q., Xue H. (2025). Optimization of echinococcosis control measures based on system dynamics. PLoS Comput. Biol..

[26] Ma W., Zhang Q., Cui Q., Duan H. (2025). Spatial-temporal distribution and influencing factors analysis of human echinococcosis in mainland china from 2004 to 2020. Acta Trop..

[27] Yang Z., Liu K., Wen B., Fu T., Qin X., Li R., Lu M., Wang Y., Zhang W., Shao Z. (2024). Changes in the global epidemiological characteristics of cystic echinococcosis over the past 30 years and projections for the next decade: Findings from the global burden of disease study 2019. J. Glob. Health.

[28] Kamali W., Wang S., Luo W., Liu S., Zhao L., Pan X., Wang B., Mu Y., Jiawuti T., Aierken K. (2024). Epidemiology and genetic diversity of *Echinococcus granulosus sensu stricto* in the East Tianshan Mountains, Xinjiang, China. Parasitol. Res..

[29] Ohiolei J.A., Xia C., Li L., Liu J., Tang W., Wu Y., Danqulamu, Zhu G., Shi B., Fu B. (2019). Genetic variation of *Echinococcus* spp. in yaks and sheep in the Tibet Autonomous Region of China based on mitochondrial DNA. Parasit. Vectors.

[30] Wei Y., Li W., Shao C., Zhao H., Hu Y., Liu H., Cao J. (2023). The polymorphic analysis of *cox1* and *cob* genes of *Echinococcus granulosus* in the Ngari region of Tibet in China. Acta Trop..

[31] Zhao Y., Gesang D., Wan L., Li J., Qiangba G., Danzeng W., Basang Z., Renzhen N., Yin J., Gongsang Q. (2022). *Echinococcus* spp. and genotypes infecting humans in Tibet Autonomous Region of China: A molecular investigation with near-complete/complete mitochondrial sequences. Parasit. Vectors.

[32] Yang Y.R., Clements A.C., Gray D.J., Atkinson J.A., Williams G.M., Barnes T.S., McManus D.P. (2012). Impact of anthropogenic and natural environmental changes on *Echinococcus* transmission in Ningxia Hui Autonomous Region, the People’s Republic of China. Parasit. Vectors.

[33] Bosco A., Alves L.C., Cociancic P., Amadesi A., Pepe P., Morgoglione M.E., Maurelli M.P., Ferrer-Miranda E., Santoro K.R., Nascimento R.R. (2021). Epidemiology and spatial distribution of *Echinococcus granulosus* in sheep and goats slaughtered in a hyperendemic European Mediterranean area. Parasit. Vectors.

[34] Alvi M.A., Ohiolei J.A., Saqib M., Li L., Tayyab M.H., Alvi A.A., Wu Y.T., Fu B.Q., Yan H.B., Jia W.Z. (2020). *Echinococcus granulosus* (*sensu stricto*) (G1, G3) and *E. ortleppi* (G5) in Pakistan: Phylogeny, genetic diversity and population structural analysis based on mitochondrial DNA. Parasit. Vectors.

[35] Odongo D.O., Tiampati C.M., Mulinge E., Mbae C.K., Bishop R.P., Zeyhle E., Magambo J., Wasserman M., Kern P., Romig T. (2018). Prevalence and genotyping of *Echinococcus granulosus* in sheep in Narok County, Kenya. Parasitol. Res..

[36] Alkhaldi A.A.M. (2024). *Echinococcus granulosus* comparative genotyping in sheep in Saudi Arabia and Egypt. Open Vet. J..

[37] Hua R.Q., Du X.D., He X., Gu X.B., Xie Y., He R., Xu J., Peng X.R., Yang G.Y. (2022). Genetic diversity of *Echinococcus granulosus sensu lato* in China: Epidemiological studies and systematic review. Transbound Emerg. Dis..

[38] Gong Q.L., Ge G.Y., Wang Q., Tian T., Liu F., Diao N.C., Nie L.B., Zong Y., Li J.M., Shi K. (2021). Meta-analysis of the prevalence of *Echinococcus* in dogs in China from 2010 to 2019. PLoS Negl. Trop. Dis..

[39] Possenti A., Manzano-Roman R., Sanchez-Ovejero C., Boufana B., La Torre G., Siles-Lucas M., Casulli A. (2016). Potential risk factors associated with human cystic echinococcosis: Systematic review and meta-analysis. PLoS Negl. Trop. Dis..

[40] Khan A., Ahmed H., Simsek S., Afzal M.S., Cao J. (2019). Spread of cystic echinococcosis in Pakistan due to stray dogs and livestock slaughtering habits: Research priorities and public health importance. Front. Public Health.

[41] Khan S., Cable J., Masud N., Hailer F., Younus M., Hussain N., Asif Idrees M., Rashid M.I., Akbar H. (2025). Epidemiological and genotypic assessment of cystic echinococcosis in ruminant populations of Northern Punjab, Pakistan: A neglected zoonotic disease. Parasitol. Res..

[42] Yu Q., Xiao N., Han S., Tian T., Zhou X. (2020). Progress on the national echinococcosis control programme in China: Analysis of humans and dogs population intervention during 2004–2014. Infect. Dis. Poverty.

[43] Fan S., Zhao X., Danqulamu, Shi B., Tang W., Dong H., Xia C. (2022). Genetic diversity and haplotype analysis of yak and sheep echinococcal cysts isolates from the mitochondrial *cox1* gene in parts of Tibet, China. Front. Vet. Sci..

[44] Yan B., Liu X., Wu J., Zhao S., Yuan W., Wang B., Wureli H., Tu C., Chen C., Wang Y. (2018). Genetic diversity of *Echinococcus granulosus* genotype G1 in Xinjiang, Northwest of China. Korean J. Parasitol..

